# Influence of Glutathione *S*-Transferase Polymorphisms on Cognitive Functioning Effects Induced by *p,p*′-DDT among Preschoolers

**DOI:** 10.1289/ehp.11303

**Published:** 2008-07-30

**Authors:** Eva Morales, Jordi Sunyer, Francesc Castro-Giner, Xavier Estivill, Jordi Julvez, Nuria Ribas-Fitó, Maties Torrent, Joan O. Grimalt, Rafael de Cid

**Affiliations:** 1 Center for Research in Environmental Epidemiology (CREAL), Barcelona, Spain; 2 Municipal Institute of Medical Research (IMIM-Hospital del Mar), Barcelona, Spain; 3 Preventive Medicine and Public Health Educational Unit, Institut Municipal d’Asistencia Sanitaria–Universitat Pompeu Fabra–Agencia de Salud Publica de Barcelona (IMAS-UPF-ASPB), Barcelona, Spain; 4 Universitat Pompeu Fabra, Barcelona, Spain; 5 CIBER Epidemiología y Salud Pública, Spain; 6 Genes and Disease Program, Centre for Genomic Regulation, Barcelona, Spain; 7 Centro Nacional de Genotipado, Barcelona, Spain; 8 Area de Salud de Menorca, IB-Salut, Menorca, Spain; 9 Departament de Química Ambiental, Consejo Superior de Investigaciones Científicas (IIQAB-CISC), Barcelona, Spain

**Keywords:** children, cognitive functioning, *p*,*p*′-DDE, *p*,*p*′-DDT, gene-environment interaction, glutathione *S*-transferase, neurodevelopment, polymorphism

## Abstract

**Background:**

Early-life exposure to *p,p*′-DDT [2,2-bis(*p*-chlorophenyl)-1,1,1-trichloroethane] is associated with a decrease in cognitive skills among preschoolers at 4 years of age. We hypothesized that genetic variability in glutathione *S*-transferase (GST) genes (*GSTP1*, *GSTM1*, and *GSTT1*) could influence the effects of prenatal exposure to *p,p*′*-*DDT.

**Methods:**

We used data from 326 children assessed in a prospective population-based birth cohort at the age of 4 years. In that study, the McCarthy Scales of Children’s Abilities were administrated by psychologists, organochlorine compounds were measured in cord serum, and genotyping was conducted for the coding variant Ile105Val from *GSTP1* and for null alleles from *GSTM1* and *GSTT1*. We used linear regression models to measure the association between organochlorines and neurodevelopmental scores by GST polymorphisms.

**Results:**

*p,p*′*-*DDT cord serum concentration was inversely associated with general cognitive, memory, quantitative, and verbal skills, as well as executive function and working memory, in children who had any *GSTP1* Val-105 allele. *GSTP1* polymorphisms and prenatal *p,p*′*-*DDT exposure showed a statistically significant interaction for general cognitive skills (*p* = 0.05), quantitative skills (*p* = 0.02), executive function (*p* = 0.01), and working memory (*p* = 0.02). There were no significant associations between *p,p*′*-*DDT and cognitive functioning at 4 years of age according to *GSTM1* and *GSTT1* polymorphisms.

**Conclusions:**

Results indicate that children with *GSTP1* Val-105 allele were at higher risk of the adverse cognitive functioning effects of prenatal *p,p*′*-*DDT exposure.

Adverse health effects due to environmental chemical exposure *in utero* and during early life have been raised as a concern in epidemiologic studies. Neonates and infants are particularly vulnerable because of their rapid growth, cell differentiation, immaturity of metabolic pathways, and development of vital organ systems (Eskenazi et al. 1999; Landrigan et al. 1999). Neurodevelopmental effects of early-life exposure to *p,p*′*-*DDT [2,2-bis(*p*-chlorophenyl)-1,1,1-trichloroethane] are among the most sensitive outcomes (Agency for Toxic Substances and Disease Registry 2000). Early-life exposure to *p,p*′*-*DDT was associated with a decrease in cognitive skills among preschoolers at 4 years of age (Ribas-Fitó et al. 2006) and with neurobehavioral dysfunction as well as impairment of mental capacities in school-age children. (Dorner and Plagemann 2002; Hardell et al. 2002). Experimental studies have shown that *p*,*p*′-DDT enhances oxidative stress and lipid peroxidation in various tissues (Koner et al. 1998; Sahoo et al. 2000). The brain is particularly susceptible to free radical–mediated insult because of its inherent biochemical and physiologic characteristics, including high lipid content and energy requirements (Pajovic et al. 2003). Reactive oxygen species are generated continuously in nervous tissue during normal metabolism and neuronal activity.

Glutathione *S*-transferases (GSTs) are a family of enzymes comprising 16 genes in six subfamilies [alpha (GSTA), mu (GSTM), omega (GSTO), pi (GSTP), theta (GSTT), and zeta (GSTZ)] that are involved in the detoxification of electrophilic intermediates, as well as lipid peroxidation produced by reactive oxygen species (Nebert and Vasiliou 2004; Strange et al. 2001). *GSTP1*, *GSTM1*, and *GSTT1* are well known to be polymorphic, and allelic variants show differences in catalytic activity. Deletion of the *GSTM1* and *GSTT1* genes, resulting in loss of functional activity, has been reported in approximately 50% and 20% of the Caucasian population, respectively (Duell et al. 2002; Hayes and Pulford 1995; Hayes and Strange 1994; Nelson et al. 1995). In GSTP1, the most strongly expressed of the GST isoenzymes in the human brain (Carder et al. 1990; Strange et al. 1992), single nucleotide substitutions at A313G result in the amino acid change Ile105Val. This variant is fairly common in Caucasians. In a healthy population, 51% were homozygous for the common allele, *GSTP1* Ile/Ile; 43% were heterozygous for *GSTP1* Ile/Val, and 6% were homozygous for the variant allele, *GSTP1* Val/Val (Harries et al. 1997). The *GSTP1* Ile105Val substitution is located near the substrate-binding site, resulting in a less active enzyme (Srivastava et al. 1999; Strange and Fryer 1999; Sweeney et al. 2000).

No studies have yet determined the relative activities of human *GSTP1*, *GSTM1*, or *GSTT1* toward organochlorines, so the potential significance of the common polymorphisms of these genes on environmental pollutants susceptibility is unknown. The aim of the present study was to investigate the relationship between cord serum levels of *p,p*′*-*DDT/*p,p*′*-*DDE [2,2-bis(*p*-chlorophenyl)-1,1-dichloroethylene] and GST gene polymorphisms as effect modifiers on infant neurodevelopment at 4 years of age.

## Population and Methods

### Study participants

The Menorca, Spain, cohort was set up in 1997 within the Asthma Multicenter Infants Cohort study (Polk et al. 2004). Menorca is a tourist island with an important agricultural sector. All women who presented for antenatal care over 12 months starting in mid-1997 were recruited. Subsequently, 482 children (94% of those eligible) were enrolled, and 470 (97.5%) provided complete outcome data up to 4 years of age. Among these children, 405 (86%) had organochlorine compounds analyzed in their cord serum, and 411 (87.4%) were genotyped. We based the present study on complete data on neurodevelopment scores, organochlorine levels, and genotype from 326 children. After explaining the study to the parents, we obtained written informed consent. This study was approved by the ethics committee of the Institut Municipal d’Investigació Mèdica.

### Study variables

Neuropsychological testing of the children at 4 years of age included assessment of intellectual and motor abilities. Two certified psychologists performed the testing, which was supervised by the project’s consulting psychologist (including intra- and interpsychologist validity at the beginning, midpoint, and end of the study). The staff involved in the neuropsychological testing did not know the degree of the child’s exposure to organochlorine compounds. Cognitive development was measured with the Spanish version of the McCarthy Scales of Children’s Abilities (MCSA) that provides information on cognitive ability and motor abilities (McCarthy 1972). The MCSA consists of 18 items derived from six different scales (assessing general cognitive, verbal, perceptual-performance, quantitative, memory, and motor abilities).

To further improve our understanding of the specific functions associated with exposure to organochlorine compounds, we reorganized the MCSA items into new outcomes according to underlying neuropsychological functions, executive function and working memory, that corresponded to traits (Kolb and Wishaw 1996). In addition, we validated executive function construct by confirmatory factor analyses, which showed an acceptable goodness of fit and a Cronbach’s alpha coefficient of 0.69 for internal consistency (Julvez et al. 2007a, 2007b). The new outcomes were thus executive function (MCSA items 2, 5, 6, 14II, 15, 17, and 18) and working memory (MCSA items 5 and 14II). The executive function involves those cognitive tasks critical to non-routine, goal-oriented situations that are performed by the prefrontal cortex, and working memory manages the information required to carry out other cognitive tasks such as learning, reasoning, and comprehension (Kolb and Wishaw 1996; Lezak and Howieson 2004).

We obtained information on socioeconomic background, maternal diseases, obstetric history, parity, child’s sex, fetal exposure to alcohol (ever exposure during pregnancy), prenatal cigarette smoking (at least one cigarette a day during the last trimester), type and duration of breast-feeding, education, and social class through questionnaires administered in person after delivery and at 48 months. We used the U.K. Registrar General’s 1990 classification to group subjects by social class according to maternal and paternal occupation coded using the International Standard Classification of Occupations (Warwick Institute for Employment Research 2006). We grouped social class into four categories according to maternal occupation (professional, skilled, nonskilled, and unemployed). We obtained information on birth weight, birth length, and gestational age through medical records. We categorized duration of breast-feeding in four groups based on quartiles (< 2, 2–15.9, 16–27.9, and ≥ 28 weeks).

For the *p*,*p*′-DDT and *p*,*p*′-DDE data we obtained for this study, a gas chromatograph with electron capture detection (Hewlett Packard 6890N GC-ECD; Hewlett Packard, Avondale, PA, USA) was used to quantify *p*,*p*′-DDT and *p*,*p*′-DDE, as described elsewhere (Carrizo et al. 2006; Sala et al. 2001). Quantification was performed by using external standards, with the polychlorinated biphenyl congener 142 (PCB-142) injection standard to correct for volume. Recovery of 1,2,4,5-tetrabromobenzene and PCB-209 (75–115%) was used to correct results. Limit of detection was 0.02 ng/mL. A value of 0.01 ng/mL was given for values below the level of detection. Serum samples were stored at −40°C until analysis. All analyses were carried out in the Department of Environmental Chemistry in Barcelona, Spain (Sala et al. 2001).

### Genotyping

We obtained DNA samples from all eligible children (*n* = 482) and included 326 in the study: 280 from blood samples (86%) and 46 from saliva samples (14%). For the DNA data we obtained, two semi-automated assays were implemented to facilitate the detection of null alleles from *GSTM1* and *GSTT1* and the coding variant Ile105Val from *GSTP1*. Detection of the *GSTM1* and *GSTT1* null alleles was performed with a method modified from that initially described by Arand et al. (1996). Briefly, a multiplex reaction with fluorescent-labeled primers was amplified in a multiplex polymerase chain reaction (PCR), and *b-globin* gene was used as a positive control for *GST* null genotypes. Expected amplicons size is 219 bp (*GSTT1*), 459 bp (*GSTM1*), and 268 bp (*b-globin*). PCR products were analyzed with an automated DNA analyzer (model ABI XL3100; Applied Biosystems, Foster City, CA, USA). Ile105Val from *GSTP1* was analyzed using the pyro-sequencing technology (Biotage, Uppsala, Sweden) in a single assay. All assays were performed blinded to the neurodevelopmental scores and organochlorine levels. *GSTP1* genotypes were in Hardy–Weinberg equilibrium in the total analyzed cohort (*p* < 0.05).

### Statistical analysis

We standardized continuous neurodevelopment outcomes to a mean (± SD) of 100 ± 15 to homogenize all the scales and facilitate interpretation of the results. We used linear regression models to measure the association of continuous normally distributed outcomes with *p,p*′*-*DDT and *p,p*′*-*DDE cord serum levels by GST polymorphisms. Because the frequency of homozygosity at the *GSTP1* Val/Val locus was relatively low, we combined the *GSTP1* Ile/Val and Val/Val genotypes as in dominant genetics models for the subsequently analyses.

We analyzed sex, school trimester at examination (the school year is separated into three trimesters, so this variable has five categories: third year, second and third trimesters; fourth year, first, second, and third trimesters), psychologist, breast-feeding, maternal social class, and maternal consumption of alcohol and use of tobacco during pregnancy as confounding variables based on *a priori* selection from previous studies on neurotoxic effects of *p,p*′*-*DDT (Ribas-Fitó et al. 2006). We used adjusted general additive models to evaluate the linearity of the relation between continuous *p,p*′*-*DDT and *p,p*′*-*DDE variables and MCSA’s cognitive outcomes through nonparametric depiction of the predictor when the effects of the other variables had been taken into account. We assessed the presence of gene–environment interactions between organochlorine exposure and *GSTP1*, *GSTM1*, and *GSTT1* polymorphisms by including interaction terms in the regression model. We conducted all statistical analyses with Stata version 8.0 statistical software (StataCorp, College Station, TX, USA).

## Results

Children included in the study had a higher birth weight (*p* < 0.04) and tended to have been breast-fed for longer periods than children who did not have complete data and who were excluded (Table 1). We observed no differences in *p,p*′*-*DDT and *p,p*′*-*DDE cord serum concentrations, whereas MCSA scores were slightly higher in children included in the study than in those excluded, although differences were not statistically significant. Mothers of the included children tended to have a higher social class (*p* = 0.11) and to smoke more during pregnancy (*p* = 0.23). Allelic frequencies of the *GSTP1*, *GSTM1*, and *GSTT1* polymorphisms did not differ between included and excluded children. The *GSTP1* genotype prevalences at polymorphic Ile105Val were 45.4% for Ile/Ile, 46.9% for Ile/Val, and 7.7% for Val/Val. The prevalences of *GSTM1* and *GSTT1* null genotypes were 57.1% and 19.0%, respectively.

*p,p*′*-*DDT concentration was inversely associated with all MCSA areas in children who had any *GSTP1* Val-105 allele, except for motor skills (Table 2). The magnitude of the effect was stronger for general cognitive (> 8-point decrement), memory, and verbal skills. Further analyses showed that *p,p*′*-*DDT was also negatively associated with executive function and working memory scores in children who had any *GSTP1* Val-105 allele. *GSTP1* polymorphisms and prenatal *p,p*′*-*DDT exposure showed a statistically significant interaction for general cognitive skills (*p* for interaction = 0.05), quantitative skills (*p* for interaction = 0.02), executive function (*p* for interaction = 0.01), and working memory (*p* for interaction = 0.02).

Moreover, *p,p*′*-*DDT cord serum concentration was inversely associated with MCSA scores, except for motor skill, in children who had *GSTM1* present allele, although without reaching statistical significance (Table 3). We found no statistically significant interactions between *p,p*′*-*DDT and *GSTM1* genotype. Associations between *p,p*′*-*DDT levels and MCSA scores for executive function and working memory did not differ according to *GSTT1* polymorphisms in children at 4 years of age (Table 3).

The adjusted associations between *p,p*′*-*DDE levels and neurodevelopmental scores at 4 years of age were weak and not statistically significantly modified for any of the *GSTP1*, *GSTM1*, and *GSTT1* polymorphisms evaluated (Table 4).

## Discussion

We observed that *GSTP1* genotype significantly modified the effects of prenatal *p,p*′*-*DDT exposure on cognitive functioning in preschoolers. Adverse effects on neurodevelopment of early-life *p,p*′*-*DDT exposure were restricted to children carrying any *GSTP1* Val-105 allele. We observed the highest effects in general cognitive, memory, and verbal skills, as well as in executive function. We found statistically significant interactions between *p,p*′*-*DDT and *GSTP1* polymorphisms for general cognitive and quantitative skills, executive function, and working memory. These effects were specific for *p,p*′*-*DDT and did not occur for *p,p*′*-*DDE. The low correlation between cord blood *p,p*′*-*DDT and *p,p*′*-*DDE (< 0.40) suggests that exposures to *p,p*′*-*DDT occurred relatively recently and thus would have been during the critical periods of neurodevelopment. Overall, the present results support the neurotoxic effects of *p,p*′*-*DDT, which, in view of GST functionalities, suggest oxidative stress as a potential mechanism.

Allelic variants of genes and genetic defects may result in a differential susceptibility toward environmental toxicants. “Low-penetrating” polymorphisms in metabolism genes tend to be much more common in the population than are allelic variants of “high-penetrating” cancer genes and are therefore of considerable importance from a public health point of view (Thier et al. 2003). The GSTs are genotypically and phenotypically polymorphic with variable genotype frequencies in different ethnic groups (Harries et al. 1997). The prevalence of genetic polymorphisms of *GSTP1*, *GSTM1*, and *GSTT1* found in our study population was similar to those previously reported from studies carried out in other European populations (Costa et al. 2006; Garcia-Closas et al. 2005; Sarmanová et al. 2000; To-Figueras et al. 1999).

No studies have yet assessed the influence of *GST* genes polymorphisms on cognitive functioning effects induced by environmental exposures. The inverse association observed between *p,p*′*-*DDT cord levels and neurodevelopment scores at 4 years of age in children with *GSTP1* Val-105 variant has not been previously reported. Isoenzymes vary in tissue distribution and level of expression during development. Cells of the embryonic nervous system express high levels of GSTP and lesser amounts of alpha and mu classes (Carder et al. 1990; Lowndes et al. 1994; Raijmakers et al. 2001; Strange et al. 1992). GSTP is strongly expressed from as early as 12 weeks gestation (Raijmakers et al. 2001) and is localized to choroid plexus, vascular endothelium, ventricular lining cells, piaarachnoid, and astrocytes. GSTP isoenzyme thus localizes to the sites of the blood–cerebrospinal fluid (CSF) barrier, blood–brain barrier, CSF–brain barrier, and piaarachnoid–brain barrier. It is ideally placed to regulate neuronal exposure to potentially toxic substances derived from blood or cerebrospinal fluid.

Gene expression so early in gestation may imply a role in protection of the developing human brain (Carder et al. 1990). Differences in specific activity and detoxification ability between GSTP1 enzymes containing Val compared with Ile at position 105 have been demonstrated with several classes of substrates (Harries et al. 1997; Johansson et al. 1998; Srivastava et al. 1999; Sweeney et al. 2000). Similarly, GSTP1 variants may also differ in detoxification of reactive oxidant damage, although this has not yet been assayed.

There are several possible explanations for the effect modification of *GSTP1* gene variants on the *p,p*′*-*DDT effect in neurodevelopment scores. First, GSTs have direct antioxidant activity (Hayes and Strange 1994); specifically, GSTP1 catalyzes the detoxification that arises from DNA oxidation (Fryer et al. 2000). *p,p*′*-*DDT enhances oxidative stress and lipid peroxidation in various tissues (Agency for Toxic Substances and Disease Registry 2000; Koner et al. 1998). Based on these mechanisms, we hypothesize that children having the *GSTP1* Val-105 variant may be more susceptible to DDT effects on neurodevelopment because of enzymatic inability to detoxify the reactive oxidant damage induced by *p,p*′*-*DDT.

*p,p*′*-*DDT has estrogenic activity, which is another potential mechanism. The central nervous system is an important target of estrogen action during development periods. Functional studies support a protective role for GSTP against estrogen-induced oxidative DNA damage (Montano et al. 2004). Having the less active *GSTP1* Val-105 variant would make brain cells in preschoolers more susceptible to estrogen-induced DNA damage by compromising the ability of GSTP enzymes to neutralize the electrophilic intermediates generated from estrogenic responses triggered by *p,p*′*-*DDT. The stronger inverse association between *p,p*′*-*DDT and cognitive function, verbal skill, and executive function found among girls in our cohort (Ribas-Fitó et al. 2006) supports this hypothesis, as well as previous findings that have reported a higher antioxidant capacity among males than females (Chen et al. 2007).

Finally, we cannot exclude that the association may be secondary to linkage disequilibrium with other variant outside the *GSTP1*. However, GSTP1 is the most strongly expressed of the GST isoenzymes in the human brain, its expression is observed as early as 12 weeks gestation, and it is highly expressed in the blood–brain barrier (Carder et al. 1990), which supports the hypothesis that *GSTP1* may be a susceptibility gene rather than a linkage disequilibrium marker.

Our study has some limitations. We did not have genotyping and exposure data from all eligible children, which made selection bias possible. However, children included in the study did not differ from nonparticipants in organochlorine levels, neurodevelopment scores, or genotyping, so any resulting effects from selection bias are likely to be minimal. In addition, small numbers of subjects in each subgroup limit the conclusions that can be made regarding interactions between organochlorine and GST genotypes in a single study. We obtained data pertaining to individual exposure and GST polymorphisms without the knowledge of neurodevelopment outcome. Consequently, exposure misclassification is assumed to be nondifferential. To our knowledge, this is the first study that analyzes the effect modification of GST polymorphisms of *p,p*′*-*DDT and *p,p*′*-*DDE influence on cognitive functioning in preschoolers. Moreover, the results can be extrapolated to other preschool populations, because this is a population of healthy children exposed to background levels of organochlorines. Finally, we did not adjust organochlorines for lipid content, but this is likely to have a weak effect in a cohort of healthy children.

In conclusion, results of the present study indicate that children with *GSTP1* Val-105 allele seem more at risk for the cognitive functioning effects of early-life *p,p*′*-*DDT exposure. Detection of different level of risk within the population and greater understanding of the etiologic mechanisms may allow for the development of new prevention strategies; thus present findings suggest that neurotoxic effects of *p,p*′*-*DDT might be mediated through oxidative stress. The clinical relevance of an 8-point decrement in the cognitive scale is of little concern; however, the population impact of this effect could be notable.

## Figures and Tables

**Table 1:** Comparison of the distribution (in percentage or mean) of child and maternal variables and genotypes of GST between included and not included preschoolers: Menorca cohort, Spain, 1997–1999.

Characteristic	Children included (*n* = 326)	Children not included^a^ (*n* = 156)	*p*-Value
Female sex (%)	50.0	45.5	0.36
Gestational age (weeks)	39.4	39.1	0.12
Birth weight (g)	3219.1	3118.5	0.04
Breast-feeding, yes (%)	84.4	78.2	0.10
Breast-feeding (weeks)			
< 2	17.8	25.6	0.19
2–15.9	26.4	25.6	
16–27.9	27.9	26.9	
≥28	27.9	21.8	
Maternal age (years)	28.9	28.9	0.89
Tobacco during pregnancy, yes (%)	22.7	17.9	0.23
Alcohol during pregnancy, yes (%)	21.2	21.5	0.82
Maternal social class (%)	(*n* = 313)	(*n* = 153)	0.11
Professional	14.7	8.5	
Skilled	52.4	49.0	
Partially skilled	14.1	17.7	
Unemployed	18.8	24.8	
MCSA score	(*n* = 326)	(*n* = 93)	
General cognitive	106.9	104.5	0.29
Perceptual performance	40.7	39.0	0.06
Memory	23.5	22.8	0.44
Quantitative	18.1	17.5	0.24
Verbal	48.2	47.9	0.80
Motor	34.7	33.5	0.15
*p*,*p*′-DDT cord serum (ng/mL)	(*n* = 326)	(*n* = 79)	
Quartile 25^b^	0.04	0.03	0.37
Mean ± SD	0.17 ± 0.23	0.20 ± 0.37	
Quartile 75^c^	0.20	0.22	
Maximum	2.28	2.09	
*p*,*p*′-DDE cord serum (ng/mL)			
Quartile 25^b^	0.58	0.50	0.72
Mean ± SD	1.63 ± 1.93	1.72 ± 2.51	
Quartile 75^c^	1.85	2.10	
Maximum	15.97	19.54	
*GSTP1* (%)	(*n* = 326)	(*n* = 85)	
Ile/Ile	45.4	43.5	0.48
Ile/Val	46.9	44.7	
Val/Val	7.7	11.8	
*GSTM1* (%)	(*n* = 318)	(*n* = 93)	
Present	42.9	41.9	0.84
Null	57.1	58.1	
*GSTT1* (%)	(*n* = 318)	(*n* = 93)	
Present	81.0	76.3	0.19
Null	19.0	23.7	

a Children with data up to the fourth-year visit but not included in the analysis because of absence of data on GST polymorphisms, neurodevelopment scores, or organochlorine levels at 4 years of age.

b Observed value at the 25% cutoff.

c Observed value at the 75% cutoff.

**Table 2:** Adjusted associations [β (SE)] between concentrations of *p*,*p*′-DDT in cord serum (ng/mL) and neurodevelopment at 4 years of age by *GSTP1* genotype: Menorca cohort, Spain, 1997–1999.^a^

	*GSTP1* genotype		
Neurodevelopment measure	Ile/Ile (*n* = 149)	Ile/Val or Val/Val (*n* = 177)	*p*-Value for interaction
MCSA area			
General cognitive	7.13 (6.16) *p* = 0.25	−8.41 (4.21) *p* = 0.04	0.05
Perceptual performance	4.67 (5.75) *p* = 0.42	−3.81 (4.15) *p* = 0.36	0.21
Memory	0.90 (6.39) *p* = 0.89	−6.75 (4.31) *p* = 0.12	0.35
Quantitative	8.96 (7.228) *p* = 0.22	−3.58 (1.46) *p* = 0.02	0.02
Verbal	0.62 (6.48) *p* = 0.92	−8.23 (4.30) *p* = 0.05	0.34
Motor	10.33 (5.62) *p* = 0.07	2.94 (4.08) *p* = 0.47	0.36
Executive function	10.17 (6.43) *p* = 0.12	−10.14 (4.24) *p* = 0.02	0.01
Working memory	7.36 (6.91) *p* = 0.29	−2.75 (1.16) *p* = 0.02	0.02

a Each cell represents outcomes from a different multivariate model, adjusted for sex, school trimester at examination, psychologist, breast-feeding, maternal social class, and maternal consumption of alcohol and use of tobacco during pregnancy.

**Table 3:** Adjusted associations [β (SE)] between concentrations of *p*,*p*′-DDT in cord serum (ng/mL) and neurodevelopment at 4 years of age by *GSTM1* and *GSTT1* genotypes: Menorca cohort, Spain, 1997–1999.^a^

	*GSTM1* genotype		*GSTT1* genotype	
Neurodevelopment measure	Present (*n* = 140)	Null (*n* = 186)	Present (*n* = 264)	Null (*n* = 62)
MCSA area				
General cognitive	−5.86 (4.67) *p* = 0.21	0.02 (4.30) *p* = 0.99	−3.10 (3.55) *p* = 0.38	−5.31 (6.67) *p* = 0.43
Perceptual performance	−3.59 (4.37) *p* = 0.41	0.10 (4.40) *p* = 0.98	−0.34 (3.50) *p* = 0.92	−4.58 (6.51) *p* = 0.48
Memory	−5.77 (4.73) *p* = 0.22	−0.001 (4.57) *p* = 1.00	−4.16 (3.65) *p* = 0.25	−3.75 (6.96) *p* = 0.59
Quantitative	−1.73 (1.79) *p* = 0.34	−3.08 (2.74) *p* = 0.26	−2.60 (1.79) *p* = 0.15	−3.29 (3.44) *p* = 0.34
Verbal	−8.15 (5.00) *p* = 0.11	1.82* (4.37) *p* = 0.68	−4.58 (3.71) *p* = 0.22	−4.15 (6.82) *p* = 0.55
Motor	4.12 (4.24) *p* = 0.33	6.49 (4.36) *p* = 0.14	5.42 (3.44) *p* = 0.12	4.44 (6.38) *p* = 0.49
Executive function	−5.30 (4.88) *p* = 0.28	−1.80 (4.37) *p* = 0.68	−4.23 (3.64) *p* = 0.25	−2.87 (7.05) *p* = 0.69
Working memory	−0.74 (1.40) *p* = 0.59	−2.29 (2.20) *p* = 0.30	−1.78 (1.46) *p* = 0.22	−1.13 (1.99) *p* = 0.57

a Each cell is a different multivariate model. Adjusted for sex, school trimester at examination, psychologist, breast-feeding, maternal social class, and maternal consumption of alcohol and use of tobacco during pregnancy.

* *p* for interaction = 0.12.

**Table 4:** Adjusted associations [β (SE)] between concentrations of *p*,*p*′-DDE in cord serum (ng/mL) and neurodevelopment at 4 years of age by GST polymorphisms: Menorca cohort, Spain, 1997–1999.^a^

	*GSTP1* genotype		*GSTM1* genotype		*GSTT1* genotype	
Neurodevelopment measure	Ile/Ile (*n* = 149)	Ile/Val or Val/Val (*n* = 177)	Present (*n* = 140)	Null (*n* = 186)	Present (*n* = 264)	Null (*n* = 62)
MCSA area						
General cognitive	−0.41 (0.57) *p* = 0.47	−0.31 (0.60) *p* = 0.61	−0.48 (0.58) *p* = 0.41	−0.09 (0.59) *p* = 0.88	−0.55 (0.44) *p* = 0.21	−0.36 (1.07) *p* = 0.73
Perceptual performance	−0.65 (0.56) *p* = 0.25	−0.83 (0.59) *p* = 0.16	−1.17 (0.54) *p* = 0.03	−0.20 (0.60) *p* = 0.74	−0.90 (0.44) *p* = 0.04	−0.84 (1.03) *p* = 0.42
Memory	−0.57 (0.58) *p* = 0.33	0.18 (0.62) *p* = 0.77	−0.12 (0.58) *p* = 0.84	0.01 (0.63) *p* = 0.98	−0.27 (0.45) *p* = 0.55	−0.32 (1.11) *p* = 0.77
Quantitative	−0.38 (0.55) *p* = 0.49	−0.12 (0.22) *p* = 0.57	−0.12 (0.23) *p* = 0.61	−0.36 (0.41) *p* = 0.38	−0.29 (0.24) *p* = 0.23	−0.26 (0.55) *p* = 0.64
Verbal	−0.32 (0.59) *p* = 0.59	0.03 (0.62) *p* = 0.96	−0.09 (0.62) *p* = 0.88	0.11 (0.60) *p* = 0.85	−0.28 (0.46) *p* = 0.55	0.003 (1.09) *p* = 0.99
Motor	0.08 (0.55) *p* = 0.88	−0.24 (0.58) *p* = 0.68	−0.75 (0.53) *p* = 0.16	0.91 (0.60) *p* = 0.13	−0.42 (0.44) *p* = 0.34	0.73 (1.01) *p* = 0.47
Executive function	−0.24 (0.59) *p* = 0.68	−0.35 (0.61) *p* = 0.57	−0.35 (0.60) *p* = 0.56	−0.20 (0.60) *p* = 0.74	−0.53 (0.45) *p* = 0.25	−0.09 (1.12) *p* = 0.93
Working memory	−0.27 (0.44) *p* = 0.54	−0.20 (0.18) *p* = 0.25	−0.16 (0.18) *p* = 0.40	−0.25 (0.34) *p* = 0.47	−0.34 (0.21) *p* = 0.11	0.10 (0.33) *p* = 0.77

a Each cell is a different multivariate model. Adjusted for sex, school trimester at examination, psychologist, breast-feeding, maternal social class, and maternal consumption of alcohol and use of tobacco during pregnancy.
